# A model for how Gβγ couples Gα to GPCR

**DOI:** 10.1085/jgp.202112982

**Published:** 2022-03-25

**Authors:** William E. McIntire

**Affiliations:** 1 Department of Molecular Physiology and Biological Physics, University of Virginia Health System, Charlottesville, VA

## Abstract

Representing ∼5% of the human genome, G-protein-coupled receptors (GPCRs) are a primary target for drug discovery; however, the molecular details of how they couple to heterotrimeric G protein subunits are incompletely understood. Here, I propose a hypothetical initial docking model for the encounter between GPCR and Gβγ that is defined by transient interactions between the cytosolic surface of the GPCR and the prenyl moiety and the tripeptide motif, asparagine–proline–phenylalanine (NPF), in the C-terminus of the Gγ subunit. Analysis of class A GPCRs reveals a conserved NPF binding site formed by the interaction of the TM1 and H8. Functional studies using differentially prenylated proteins and peptides further suggest that the intracellular hydrophobic core of the GPCR is a prenyl binding site. Upon binding TM1 and H8 of GPCRs, the propensity of the C-terminal region of Gγ to convert into an α helix allows it to extend into the hydrophobic core of the GPCR, facilitating the GPCR active state. Conservation of the NPF motif in Gγ isoforms and interacting residues in TM1 and H8 suggest that this is a general mechanism of GPCR–G protein signaling. Analysis of the rhodopsin dimer also suggests that Gγ–rhodopsin interactions may facilitate GPCR dimer transactivation.

## Teaser

The heterotrimeric G protein contains an α subunit and βγ dimer, which relay signals from G-protein-coupled receptors (GPCRs) to the intracellular milieu. McIntire proposes a model in which initial interactions between Gβγ and GPCR prime the receptor for subsequent interactions with Gα.

## Introduction

It has been 40 years since the α, β, and γ subunits of heterotrimeric G proteins, which link GPCRs to intracellular effectors, were first characterized (Northup et al., 1980; Fung et al., 1981; Hildebrandt et al., 1984). Although much of the initial research focused on the Gα subunit as the central figure in GPCR–G protein interactions, Gβγ was later shown to increase the affinity of transducin α for rhodopsin and plays a critical role in nucleotide exchange (Phillips et al., 1992). In particular, the prenylated C-terminal tail of Gγ was identified as a determinant in GPCR–G protein coupling (Kisselev et al., 1994; Yasuda et al., 1996). With these facts in mind, the crystal structure of Gs bound to the β_2_ adrenergic receptor was solved in 2011 (Rasmussen et al., 2011), while a milestone in our understanding of GPCR–Gα interactions, revealed no details of interactions between GPCR and Gγ. Since these interactions may be transient and thus difficult to study using current techniques in structural biology, I have used the structural homology between the NPF binding protein Eps15 and GPCRs to construct a hypothetical model for the initial interactions between GPCR and Gβγ, followed by Gα, in a sequential fit mechanism. Although GPCR oligomerization has been intensively investigated and is clearly important for the regulation of GPCR signaling (Fotiadis et al., 2006), the model presented here will address a monomeric GPCR. Nevertheless, this model could also be applicable to multimeric GPCRs, as monomeric GPCRs have been proposed and demonstrated to be the minimal functional unit necessary to couple to G proteins (Chabre and le Maire, 2005; Whorton et al., 2007).

## Methods

In Fig. 2, the Gγ sequences were first aligned with Clustal Omega 2.1, and then two residues of the N-terminal and the C-terminal regions after the NPF motif were manually aligned. Interactions between residues of different protein chains in Fig. 3, 5, 6, 8, 9, 10, and 11, either depicted as solid lines between sequences or dashed yellow lines in structures, were determined using PyMOL to select contacts between chains <4 Å. To determine interactions within the rhodopsin chain in Fig. 8, both PyMOL and PIC (Protein Interactions Calculator), Molecular Biophysics Unit, Indian Institute of Science, Bangalore, were used (Tina et al, 2007). In Fig. 4, TM1, ICL1, and H8 sequences from class A GPCRs were compiled using www.gpcrdb.org (Pandy-Szekeres et al., 2018; Munk et al., 2019); multiple sequence alignments were generated using Weblogo3. In Figs. 5, 6, and 9, the αC and αB region of the EH_2_ domain were manually aligned with H8 and TM1 of rhodopsin, respectively. EH_2_ peptide structures (PDB accession nos. 1FH8 and 1FF1) and the rhodopsin dimer in Fig. 9 (PDG accession no. 6OFJ) contain hydrogens, unlike the structure of rhodopsin–Gt α peptide in Fig. 6 (PDB accession no. 3DQB), rhodopsin in Fig. 8 (PDB accession no. 1U19), and rhodopsin–Gi in Fig. 10 (PDB accession no. 6QNO). Thus, this analysis results in an apparently larger number of interactions between EH_2_ domains and NPF-containing peptides and the rhodopsin dimer than rhodopsin and G protein subunits in Figs. 6, 8, and 10. PyMOL was used to align structures with high homologies, such as the R- and T-states of Gβγ in Fig. 10. It should be noted that for certain objects, only a general binding area is predicted. For example, this model predicts that the farnesyl moiety binds to the hydrophobic core of rhodopsin, but the exact location is unclear, thus the position of farnesyl in Fig. 11 C is somewhat arbitrary. Related to this uncertainty is the position of the elongated Gγ_1_ h2 helix in Fig. 11 D. Although the residues at positions H2.20, H2.21, and H2.22 in Gγ that anchor the N-terminus of the elongated h2 helix are known, the position of the C-terminus of the elongated h2 helix in Fig. 11 D is also arbitrary as it depends on the position of the farnesyl moiety.

### Integration of model into GPCR–G protein activation cycle

**Figure 1.**
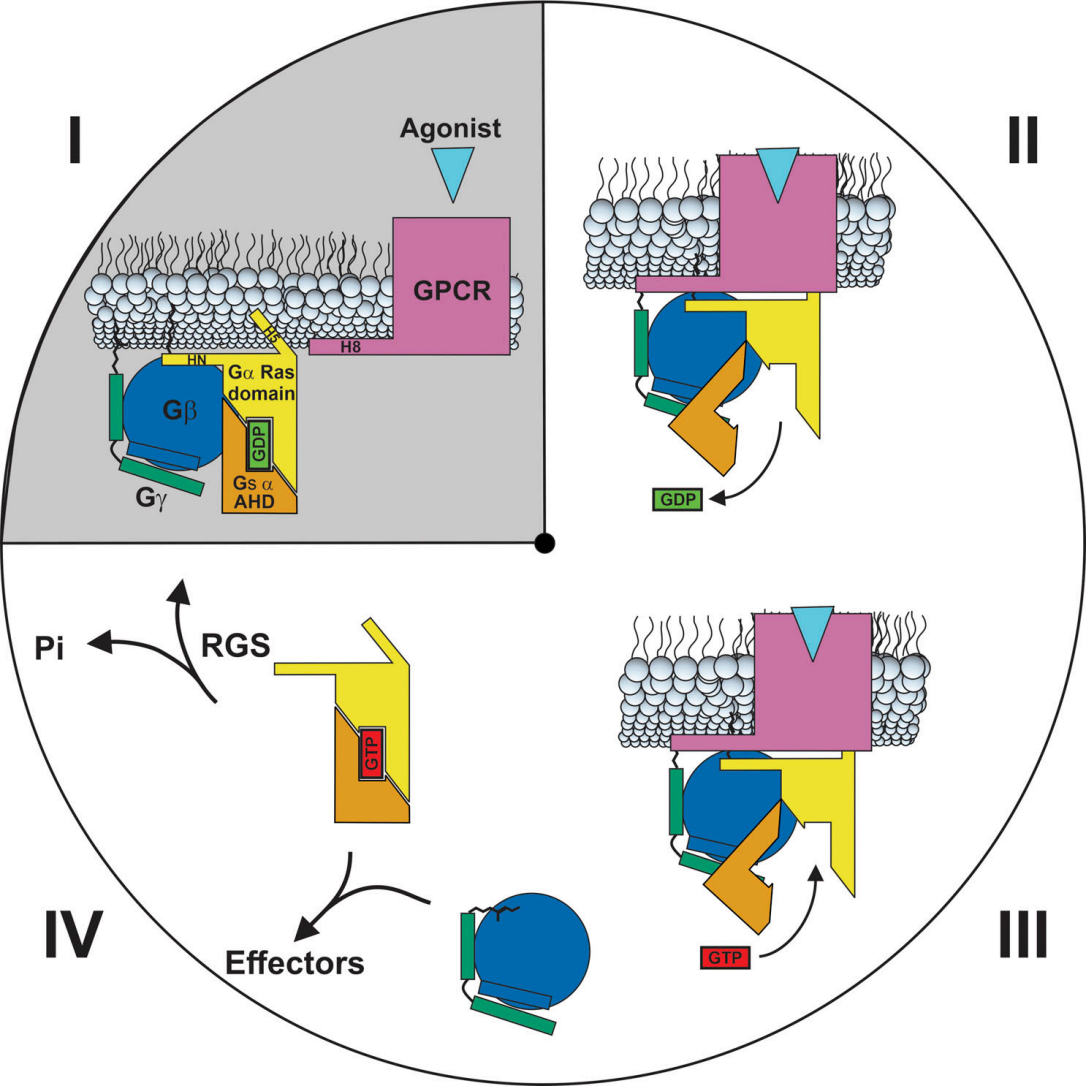
**GPCR–G protein activation cycle.** (I) Membrane-bound GDP bound Gα:βγ adjacent to GPCR, prior to R–G complex formation. (II) Interaction between agonist bound GPCR and G protein, with C-terminus of Gα inserted into the GPCR hydrophobic core. GPCR and Gβγ catalyze the release of GDP. (III) Binding of GTP to Gα induces a conformational change resulting in dissociation of Gα and Gβγ from each other and from GPCR. (IV) Gα and Gβγ regulate membrane-bound and cytosolic effectors. Intrinsic GTPase activity of Gα, aided by RGS proteins, causes GDP bound Gα to reassociate with Gβγ and relocate to the membrane as in I to repeat the cycle. The grey area indicates the points in the cycle which are addressed by the model.

Fig. 1 illustrates a simplified cartoon of the GPCR–G protein activation cycle; the model proposed here relates to the step in Fig. 1, I (grey quadrant), where the prenylated C-terminus of Gβγ makes an initial encounter with TM1, ICL1, and H8 of the GPCR. Fig. 1, II illustrates a progression of the cycle, with GPCR complexed with G protein in the high-affinity nucleotide-free state. In the next step (Fig. 1, III), GTP binds to Gα, and Gα and Gβγ separate from each other and GPCR to regulate effectors (Fig. 1, IV). The intrinsic GTPase activity, as well as RGS proteins convert GTP to GDP, facilitating the reformation of the heterotrimeric G protein in Fig. 1, I. Structures exist for heterotrimeric G proteins, individual G protein subunits, GPCRs, and GPCR–G protein complexes; however, there is a dearth of structural evidence that would shed light on a mechanism for the initial encounter between Gβγ and GPCR. This model proposes molecular details of such an initial encounter and a different orientation between GPCR and Gβγ that would facilitate these interactions.

### A proposal for a common numbering system for human Gγ isoforms

**Figure 2.**
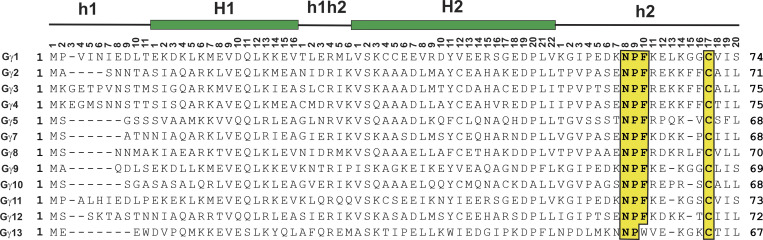
**Alignment of human Gγ isoforms.** Accession numbers used for Gγ isoforms: Gγ_1_, NG_051196.1; Gγ_2_, NM_053064.5; Gγ_3_, AF493871; Gγ_4_, AF493872.1; Gγ_5_, AF493873.1; Gγ_7_, AF493874.1; Gγ_8_, AF493875.1; Gγ_9_, AF493876.1; Gγ_10_, AF493877.1; Gγ_11_, AF493878.1; Gγ_12_, AF493879.1; and Gγ_13_, AF493880.1. Secondary structural elements are indicated by solid black lines and lowercase letters for random coil, and solid green bars and uppercase letters for α-helices; the position of residue in each secondary structural element is shown across the top of the alignment.

Gγ subunits have several highly conserved motifs that are important for coupling to GPCRs, including the NPF motif in the C-terminal region and the C-terminal cysteine, which is the target for either farnesylation or geranylgeranylation. Fig. 2 illustrates the alignment of the 12 human Gγ isoforms with the NPF motif and C-terminal cysteine highlighted in a yellow background. This alignment will serve as the basis for a novel common Gγ numbering system (CGγN) for human isoforms based on the CGN system implemented for Gα isoforms in Flock et al. (2015). The secondary structure of two α helices (H1 and H2), which are separated by a hinge or loop (h1h2) region and flanked by N-terminal and C-terminal random coils (h1 and h2, respectively), is based on crystal structures of Gγ_1_ and Gγ_2_, the only isoforms that have been solved to date. The CGγN uses a similar nomenclature as the CGN, including the secondary structure and position; however, instead of a domain descriptor in the CGN system, the CGγN system will simply begin with Gγ to differentiate it from the CGN system. Based on Fig. 2, for example, the proline in the NPF motifs of Gγ_1_ and Gγ_2_ would be Gγ_1_: Pro63^Gγh2.9^ and Gγ_2_: Pro60^Gγh2.9^, respectively. This nomenclature will simplify the discussion of various residues in human Gγ isoforms and likely has applicability to other species, as high homology in Gγ isoforms in mammals and amphibians has been observed (Cook et al., 2001).

### TM1, ICL1, and H8 form an NPF binding site in GPCRs

**Figure 3.**
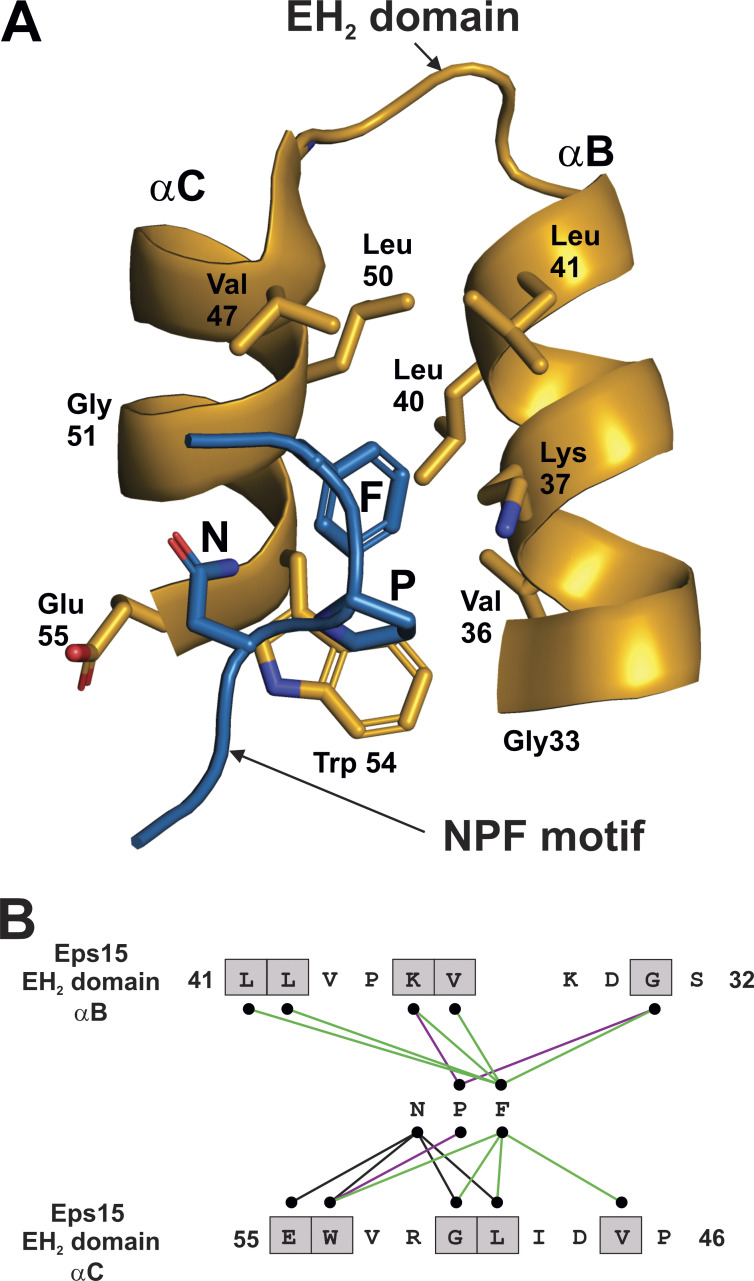
**NPF interaction with EH**_**2**_ **domain in Eps15.** **(A)** αB and αC helices (orange) and NPF motif (blue) from bound peptide PTGSSSTNPFR from the EH_2_ domain of human EPS15 (PDB accession no. 1F8H). **(B)** Residues from the αB and αC helices that contact the NPF motif are indicated in grey boxes; interacting residues were determined in PyMOL as any contact <4 Å. Contacts between Asparagine of NPF and αB and αC helices are indicated by black lines; contacts between proline of NPF and αB and αC helices are indicated by purple lines; contacts between phenylalanine of NPF and αB and αC helices are indicated by green lines.

**Figure 4.**
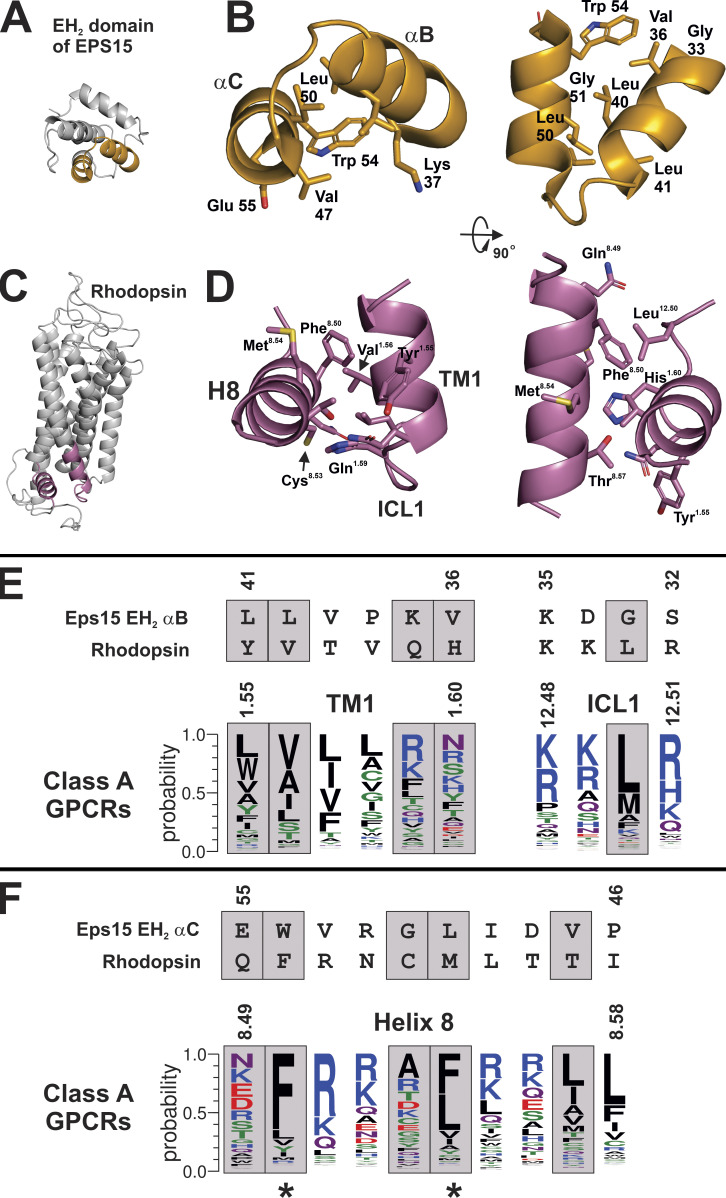
**Structural homology between αB and αC helices of EH**_**2**_ **and TM1, ILC1 and H8 of rhodopsin.** **(A)** αB and αC helices from the EH_2_ domain are highlighted in orange. **(B)** Closeup of αB and αC helices from A showing NPF interacting side chains. **(C)** Ground state structure of rhodopsin (PDB accession no. 1U19) with TM1, ICL1, and H8 in reddish-purple. **(D)** Closeup of TM1, ICL1, and H8 from C. Rhodopsin was oriented to emphasize homology in secondary structure between H8 and the αC helix, and TM1 and the αB helix in B. **(E)** The sequence of the αB helix from the EH_2_ domain in B was aligned manually with the sequence of TM1 and ICL1 from rhodopsin in D and multiple sequence alignments from class A GPCRs. Grey boxes indicate residues in the EH_2_ domain that contact the NPF motif as shown in Fig. 3 B; aligning residues in GPCRs are also in grey boxes. Polar residues Gly, Ser, Thr, Tyr, and Cys are green; neutral residues Gln and Asn are purple; basic residues Lys, Arg, and His are blue; acidic residues Asp and Glu are red; and hydrophobic residues Ala, Val, Leu, Ile, Pro, Trp, Phe, and Met are black. **(F)** The sequence of the αC helix from the EH_2_ domain in B was aligned manually with the sequence of H8 from rhodopsin in D and multiple sequence alignments from class A GPCRs. Grey boxes indicate residues in the EH_2_ domain that contact the NPF motif as shown in Fig. 3 B; aligning residues in GPCRs are also in grey boxes. Residues in the multiple sequence alignment are colored as in E. Asterisks under multiple sequence alignments indicate residues in the αC helix of the EH_2_ domain that are most critical for interactions with the NPF motif, and by analogy, the residues in H8 that may be most important for interactions with the NPF motif in Gγ.

The NPF motif in the Gγ_1_ isoform (positions h2.8, h2.9 and h2.10, Fig. 2) has been shown to be critical for productive interactions between Gt and rhodopsin (Kisselev and Downs, 2006). To look for a mechanistic explanation for how the NPF motif facilitates Gβγ–GPCR interactions, other NPF binding proteins were examined. One protein containing the NPF binding sites is Eps15, originally identified as a substrate for the EGFR (Fazioli et al., 1993), but later also revealed to contain protein binding domains conserved across plants, fungi, and animals, referred to as Eps15 homology (EH) domains (Paoluzi et al., 1998). The EH domain is a signaling module that is comprised of two EF hands, resulting in a structure with four closely associated α helices (Confalonieri and Di Fiore, 2002), two of which, αB and αC, intersect to form a binding pocket for short amino acid motifs such as NPF, HT/SF, WW, or FW (Paoluzi et al., 1998). The solution structures for the second EH domain of Eps15 (EH_2_) bound to two different NPF containing peptides were solved by NMR (de Beer et al., 2000) and revealed the basis for NPF motif–EH domain interactions. Fig. 3 A shows the interactions between the peptide ST**NPF**R, which forms a type I Asn-Pro β-turn structure, and the EH_2_ domain of Eps15 (de Beer et al., 2000); this structure was chosen because the peptide closely resembles the NPF motif and surrounding residues in many of the Gγ isoforms, especially Gγ_5_, which has the same sequence from residues h2.6–h2.11 (Fig. 2). Mutational analysis of an NPF containing peptide has demonstrated that each of the residues in the NPF motif is required for binding to EH domains in Eps15 (Salcini et al., 1997). The relationship between the αC and αB regions of the EH_2_ domain and the NPF containing peptide is shown in Fig. 3 A. Contacting residues between EH_2_ and the NPF motif of the peptide are indicated by lines in Fig. 3 B; these interactions illustrate that phenylalanine of NPF serves as a hydrophobic anchor, binding deep in the cleft formed by the αB and αC helices. The most critical elements of the NPF binding site, common to all EH domains, are the highly conserved tryptophan and leucine in the αC helix (Trp54 and Leu50 of EH_2_ in Eps15), and mutation of either residue to alanine abrogates NPF binding (Paoluzi et al., 1998; Fig. 4 F, asterisks).

In using the structure of the STNPFR peptide bound to the EH_2_ domain as a model for Gγ–GPCR interactions, the peptide is the structural correlate of the NPF region of Gγ, but the structural correlate to the NPF interacting site of the EH_2_ domain in rhodopsin was not immediately clear. However, biochemical studies offered several clues as to the NPF binding site in rhodopsin. For example, the surface of H8 of rhodopsin (Ernst et al., 2000), and in particular Cys316^8.53^ (Downs et al., 2006), has been shown to be the contact site for the prenylated C-terminus of Gγ_1_ (superscript refers to Ballesteros–Weinstein numbering system; Ballesteros, 1995). It is also noteworthy that Cys316^8.53^ of rhodopsin is adjacent to and interacts with TM1 in less than fully active structures of rhodopsin, such as the ground state (PDB accession no. 1U19) and a photoactivated deprotonated intermediate state (Salom et al., 2006; PDB accession no. 2I37). Conversely, the residues at the C-terminus of G_γ1_ that were most important for rhodopsin interactions were revealed to be Asn62^Gγh2.8^, Pro63^Gγh2.9^, and Phe64^Gγh2.10^ (Kisselev and Downs, 2006). Taken together, these studies suggest that the region of H8 in rhodopsin adjacent to TM1 is a likely binding site for the NPF region of G_γ1_. Since the Gγ NPF motif contacts Gβ in all known structures of Gβγ, alone or in complex with other proteins, the biochemical evidence suggests an alternative conformation in which the Gγ NPF motif contacts rhodopsin, which will be described in the following model.

The structural relationship between the αC and αB helices of the EH_2_ domain of Eps15 (Fig. 4 A, orange) echoes the interaction of H8 and TM1, respectively, with rhodopsin (Fig. 4 C, reddish-purple). Colored regions are enlarged in Fig. 4 B (EH_2_) and Fig. 4 D (rhodopsin). A gross comparison of Fig. 4, B and D reveals a conservation in the secondary and tertiary structure, with intersecting helices present in both structures. Alignment of the tertiary structures of TM1/H8 domains and the EH_2_ domain allows the analysis of individual residues that are in analogous positions. Interestingly, a rotation of H8 clockwise ∼115° from the perspective of the distal end of H8 would most closely align the residues of H8 with the homologous NPF binding residues of the αC helix. Residues from the TM1 and ICL1 region of rhodopsin were aligned with residues of the αB helix of the EH_2_ domain of Esp15 in Fig. 4 E, based on the superposition of helices from the EH_2_ domain and rhodopsin in Fig. 4, B and D. H8 of rhodopsin was similarly aligned with the analogous sequence from the αC helix of the EH_2_ domain of Esp15 in Fig. 4 F. Residues in the EH_2_ domain that make contact with the NPF motif, along with the corresponding residues in rhodopsin, are indicated with a grey box (Fig. 4, E and F). Conservation of rhodopsin residues (Fig. 4, E and F) among Class A GPCRs is shown with the rhodopsin TM1 and ICL1 domains (Fig. 4 E) and H8 domain (Fig. 4 F).

Many of the hydrophobic NFP binding residues in the EH_2_ domain (Fig. 4, E and F) are also present in the analogous positions in rhodopsin and conserved in class A GPCRs. Most importantly, the essential Trp54 and Leu50 of EH_2_ in Eps15 are mirrored in the analogous Met^8.54^ and Phe^8.50^ of rhodopsin, respectively (Fig. 4 F, marked with asterisks). Residues at the positions 8.50 and 8.54 are almost always hydrophobic, typically phenylalanine or leucine, in class A GPCRs (Fig. 4 F). Also, Leu40 and Leu41 of the αB helix of EH_2_ are similarly hydrophobic to Val^1.56^ and Tyr^1.55^ in rhodopsin, respectively (Fig. 4 E). Residues at positions 1.55 and 1.56 are also typically hydrophobic in class A GPCRs (Fig. 4 E). Although Thr8.57 does not match the hydrophobicity of Val47 of the αC helix of EH_2_ (Fig. 4 F), residues in class A GPCRs at this position are typically hydrophobic. Interestingly, the glycine at position 33 in the αB helix of EH_2_ does not align with anything in TM1 since the TM1 helix ends; however, Leu^12.50^ of rhodopsin appears to form a part of the putative NPF binding site (Fig. 4, C and D). Hydrophobicity is conserved at position 12.50 of ICL1 in class A GPCRs, with leucine or methionine being the most common residues. Electrostatic interactions also appear to be conserved between NPF binding sites in the EH_2_ domain and the TM1/H8 domain. For example, Lys37 in the αB helix of EH_2_ corresponds to the polar Gln^1.59^ in TM1 of rhodopsin, as well as arginine or lysine, which tend to be prevalent at this position in class A GPCRs (Fig. 4 E). Further, Glu55 in the αC helix of EH_2_ aligns with Gln^8.49^ of rhodopsin (Fig. 4 F); the residue at this position in class A GPCRs is usually charged or polar. These elements of homology, in conjunction with biochemical evidence described above, suggest that the pocket formed by TM1, ICL1, and H8 forms an NPF binding site in class A GPCRs that is involved in the initial interactions between GPCR and Gβγ. Since ESP15 is functionally unrelated to GPCRs, there may be differences in how EH_2_ domains and GPCRs interact with NPF-containing proteins. One of the main differences is the requirement of GPCRs for NPF-containing peptides (the Gγ subunit) to be prenylated, whereas ESP15 can interact productively with NPF-containing peptides lacking prenylation. This prerequisite of prenylation for Gγ subunits to productively interact with GPCRs suggests that the mode of binding of NPF-containing proteins may be distinct between GPCRs and EH_2_ domains. Notwithstanding these differences, structures of EH_2_ domains binding to NPF-containing peptides represent the best available model for understanding the interaction between the NPF-containing region of Gγ and GPCRs.

### Residues adjacent to the NPF motif may confer specificity in GPCR–Gβγ interaction

**Figure 5.**
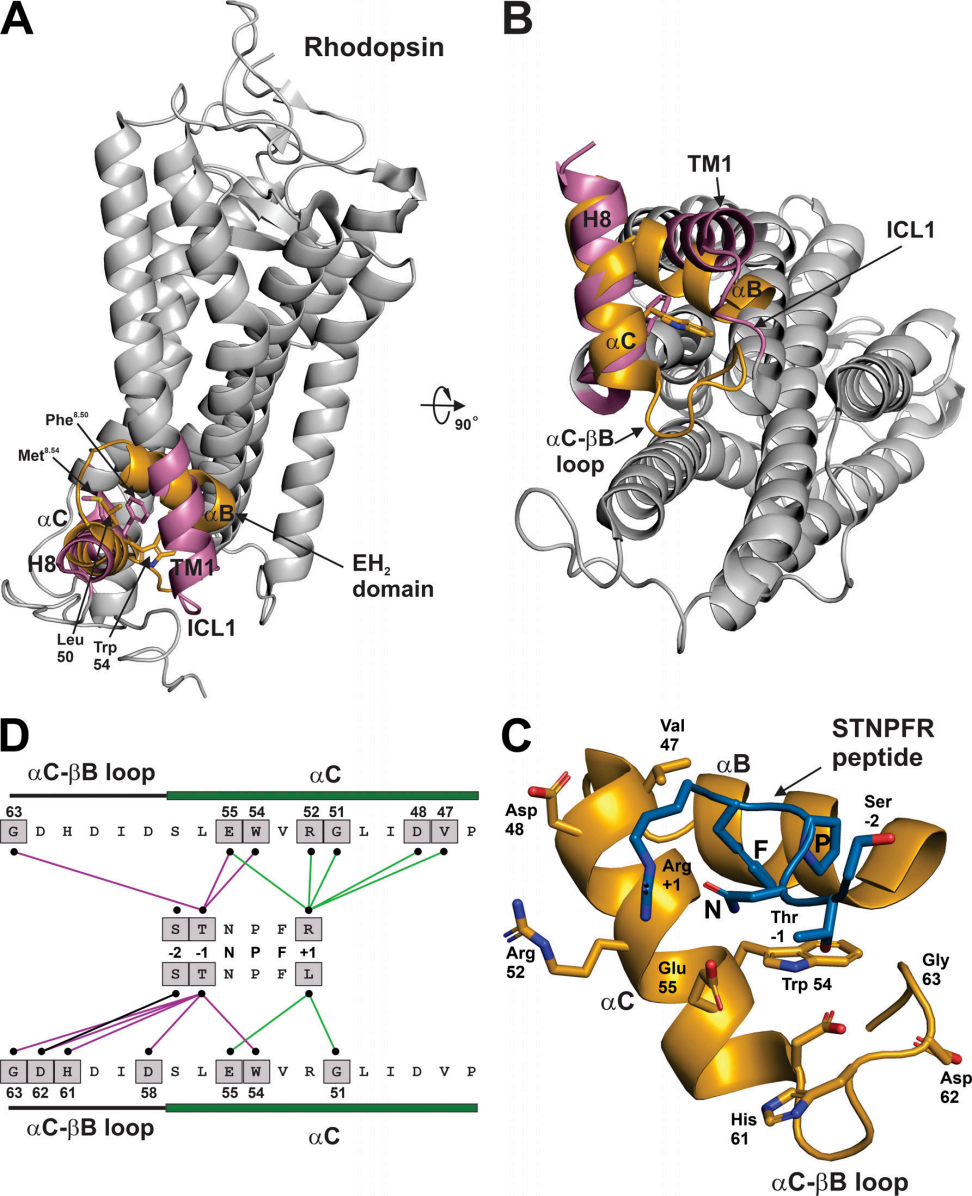
**Interactions between NPF surrounding residues and the EH**_**2**_ **domain.** **(A)** The αB and αC helices of EH_2_ were manually aligned with TM1 and H8 of rhodopsin, respectively. **(B)** Rotation of A by 90° to visualize the Gt binding surface of rhodopsin. Note that ICL1 of rhodopsin occupies a similar space as the αC–αB loop of the EH_2_ domain. **(C)** Closeup of B with rhodopsin removed and the STNPFR peptide shown in its binding orientation with the EH_2_ domain. **(D)** Comparison of the EH_2_ domain contacts with residues surrounding the NPF motif in the structures of the EH_2_ domain bound to PTGSSSTNPFR (PDB accession no. 1F8H) and the EH_2_ domain bound to PTGSSSTNPFL (PDB accession no. 1FF1). Residues from the αC–αB loop and αC helix that contact the residues surrounding the NPF motif are indicated in grey boxes; interacting residues were determined in PyMOL as any contact <4 Å. Contacts between residues at the −2 position, with respect to NPF, and αC–αB loop and αC helix are indicated by black lines; contacts between residues at the −1 position, with respect to NPF, and αC–αB loop and αC helix are indicated by purple lines; contacts between residues at the +1 position, with respect to NPF, and αC–αB loop and αC helix are indicated by green lines.

Although many EH domain-containing proteins bind the NPF motif, there is clearly a binding specificity between the identity of residues surrounding the NPF motif and the EH domain, even between different EH domains in the same protein. For example, phage display was used to show that the EH_1_ and EH_3_ domains of the mammalian protein Eps15R, and the EH_3_ domain of the yeast protein YBL047C prefer binding peptides with an arginine at the +1 position with respect to the NPF motif (Paoluzi et al., 1998); this appeared to be a structural requirement as well as a chemical requirement, as lysine at the +1 position was not observed in any of the peptides. The interactions between the serine, threonine, and arginine or leucine residues surrounding the NPF motif and the residues in the αB and αC helix of the EH_2_ domain of Eps15 are compared in Fig. 5. Alignment of αC and αB of the EH_2_ domain with H8 and TM1 of rhodopsin, respectively, is shown in Fig. 5 A. Rotation of Fig. 5 A 90° away from the viewer illustrates the Gt-binding surface of rhodopsin (Fig. 5 B). Fig. 5 C shows a closeup of the EH_2_ domain from Fig. 5 B, with residues in the EH_2_ domain that make contact with residues surrounding the NPF motif labeled. Fig. 5 D compares the contacts between the EH_2_ domain and the STNPFR and STNPFL peptides. One important point is that the αC–αB loop of the EH_2_ domain, which contacts the serine and threonine of the STNPFL peptide, has ICL1 as a structural analog in rhodopsin (Fig. 5, A and B), which may work with TM1 and H8 to influence binding to specific Gγ isoforms.

For example, the arginine at position +1 in the ST**NPF**R peptide was postulated to contribute to the stability of the Asn-Pro β-turn (de Beer et al., 2000) through interactions with residues Val47, Asp48, (Gly51), Arg52, and Glu55 in the αC helix (Fig. 5 D), thus strengthening the interaction between NPF motif and EH domain. This result was shown to be true experimentally, as the STNPFR peptide was shown to have a higher affinity for the EH_2_ domain of Eps15 than the same peptide in which the arginine was replaced with a leucine (de Beer et al., 2000). Interestingly, a leucine to alanine mutation at the +1 position of the SSST**NPF**L peptide from RAB was shown to diminish binding to a GST fusion protein containing the three EH domains from Esp15 (Salcini et al., 1997). This may explain in part why a farnesylated Gγ_1_ C-terminal peptide with residues h2.11 and h2.12 reversed, resulting in glutamic acid at the +1 position, failing to stabilize the active form of rhodopsin (Kisselev and Downs, 2003).

These modulating effects of different residues at the +1 position relative to NPF on binding EH domains have implications for Gγ isoforms, as Gγ_1_, Gγ_7_, Gγ_9_, Gγ_11_, and Gγ_12_ have a lysine at the +1 position (h2.11; Fig. 2), while Gγ_2_, Gγ_3_, Gγ_4_, Gγ_5_, Gγ_8_, and Gγ_10_ have an arginine at the h2.11 position; Gγ_13_ appears to be an outlier with a valine at the h2.11 position. The same phage display technique found that the EH_2_ domain of the yeast protein PAN1 preferred the consensus sequence NPFxD (Paoluzi et al., 1998). This consensus sequence could contribute to specificity in GPCR Gβγ interactions, as Gγ_7_, Gγ_8_, and Gγ_12_ contain the NPFxD motif, with aspartic acid at the h2.12 position, while Gγ_1_, Gγ_2_, Gγ_3_, Gγ_4_, Gγ_9_, Gγ_10_, Gγ_11_, and Gγ_13_ have a similar NPFxE motif, with glutamic acid at position h2.12, and Gγ_5_ has a proline at position h2.12 (Fig. 2).

The residues preceding the NPF motif have also been shown to influence interactions with EH domains. For instance, a serine to alanine mutation at the −2 position and a threonine to alanine at the −1 position of the SSST**NPF**L peptide from RAB diminished binding of the peptide to a GST fusion protein containing the EH domains from Eps15, with the mutation at position −1 having the larger effect (Salcini et al., 1997). The effect of these mutations is not completely unexpected, as the residues at the −1 and −2 positions in the ST**NPF**R make contact with residues in the αC helix of the EH_2_ domain of Eps15 (Fig. 5 D). This may explain the specificity observed in which a prenylated C-terminal Gγ_5_ peptide with a STNPFR motif (Fig. 2) was found to inhibit M_2_ and M_4_ muscarinic signaling, whereas prenylated C-terminal peptides from Gγ_7_ and Gγ_12_ with a SENPFK motif (Fig. 2) were not able to inhibit M_4_ signaling (Azpiazu et al., 1999). Further, a prenylated C-terminal Gγ_5_ peptide was found to stabilize a unique state of the M_2_ receptor with greater efficacy than the analogous peptides from Gγ_2_, Gγ_7_, or Gγ_12_ (Azpiazu and Gautam, 2006). The residues at the −1 position with respect to the NFP motif in Gγ isoforms may explain the specificity of Gγ_5_ at the M2 receptor. In the Gγ_5_ isoform, the residue at the −1 position is relative to the NPF motif is Thr56^h2.7^ (Fig. 2), which has been shown to positively influence NPF binding to EH domains (Salcini et al., 1997); in contrast, the residue at position h2.7 in Gγ_2_, Gγ_7_, and Gγ_12_ is glutamic acid (Fig. 2). Interestingly, Gγ_5_ peptides were not able to inhibit α_2_-adrenergic, somatostatin, or M1 signaling (Azpiazu et al., 1999).

Specific residues in the EH domain can also affect NPF–EH domain interactions. For example, in a study of different EH domains, it was found that the residue at the +3 position relative to the conserved tryptophan, Trp54 in the EH_2_ domain of Eps15, analogous to position 8.47 in H8 of GPCRs, was shown to contribute to the affinity of the domain for NFP motifs, and to modulate the specificity of binding depending on the residues adjacent to the NFP motif (Paoluzi et al., 1998). Perhaps most intriguing, analysis of the +1 residues in Fig. 5 D indicates that this residue can affect the binding of residues at the −1 and −2 positions, as the serine and threonine make more extensive interactions with αC–αB loop when the +1 residue is a leucine than when it is an arginine. Thus, the surrounding structural elements of the NFP motif in Gγ isoforms and H8-TM1-ICL1 NPF binding pocket in the GPCRs may be involved in the regulation of GPCR–Gβγ interactions.

### How Gγ CT–GPCR affinity relates to R–G coupling efficiency

Regarding the issue of how affinity between GPCR and the C-terminus of Gγ affects R–G coupling, the assumption that affinity of a Gγ C-tail is positively correlated to the function of the Gγ with respect to GPCR coupling may lead to confusion. For example, a scrambled Gγ_5_ C-terminal peptide was found to interact less effectively with the M2 muscarinic receptor than the wild type Gγ_5_ C-terminal peptide (Azpiazu et al., 1999); however, a β_1_γ_5_ dimer containing the scrambled Gγ C-terminal sequence was more efficient than the wild type β_1_γ_5_ dimer at catalyzing nucleotide exchange at the M2 receptor (Azpiazu and Gautam, 2001). The scrambled β_1_γ_5_ dimer appeared to have some level of residual activity as it could form heterotrimers with Go α and activate PLCβ_3_, whereas the replacement of the NPFR motif with AAAA or truncation of 10 residues before the cysteine at position h2.17 in Gγ_5_ abolished these functions. This result may appear paradoxical, but it is consistent with the observation that the β_1_γ_2_ dimer exhibited a higher affinity for rhodopsin than the physiologically relevant dimer, β_1_γ_1_ (Jian et al., 2001). Thus, a Gβγ dimer with high catalytic activity interacts rapidly and transiently with GPCRs.

### Evidence for a specific farnesyl binding site in rhodopsin

Several studies have suggested that interactions between rhodopsin and the farnesylated proteins Gγ_1_ and GRK1 are mediated in part by a specific farnesyl docking site in rhodopsin. For example, an S-prenylated cysteine analog was shown to inhibit the ability of light-activated rhodopsin to catalyze nucleotide exchange in Gt; this inhibition could be overcome by increasing the amounts of activated rhodopsin or Gt βγ, suggesting a direct interaction of the Gγ_1_ farnesyl moiety and rhodopsin (Scheer and Gierschik, 1995). This direct interaction was later confirmed using a photoreactive farnesyl analog incorporated into the C-terminal cysteine of γ_1_ in Gt βγ; after reconstitution with Gt α and rhodopsin into membranes, the farnesyl analog was specifically crosslinked to the light-activated rhodopsin (Katadae et al., 2008). Specificity of the interaction between the farnesyl moiety of Gγ_1_ and rhodopsin was demonstrated with the finding that C-terminal Gγ_1_ peptides that were geranylgeranylated were less efficacious than the same farnesylated peptides at stabilizing the light-activated form of rhodopsin (Kisselev et al., 1995a). This was an important observation, for if the interaction between rhodopsin and prenyl moiety was based strictly on hydrophobicity, the more hydrophobic geranylgeranylated peptides would have been expected to be at least as efficacious as the farnesylated peptides. A similar study reported that the replacement of the farnesyl group of GRK1 with geranylgeranyl reduced the ability of GRK1 to interact with rhodopsin, although the geranylgeranylated GRK1 was still able to associate with membranes; this result led the authors to reason that there was a specific farnesyl docking site on rhodopsin (Inglese et al., 1992). Evidence that the farnesyl binding site on rhodopsin used by Gγ_1_ and GRK1 may in fact be the same site was provided by a study that demonstrated inhibition of rhodopsin kinase activity by a farnesylated Gγ_1_ C-terminal peptide, as well as farnesylated GRK1 C-terminal peptides (McCarthy and Akhtar, 2000). Taken together, these data suggest a common farnesyl binding site on rhodopsin that is critical for the initial interactions of both GRK1 and Gγ_1_.

### The intracellular GPCR core as prenyl binding site

**Figure 6.**
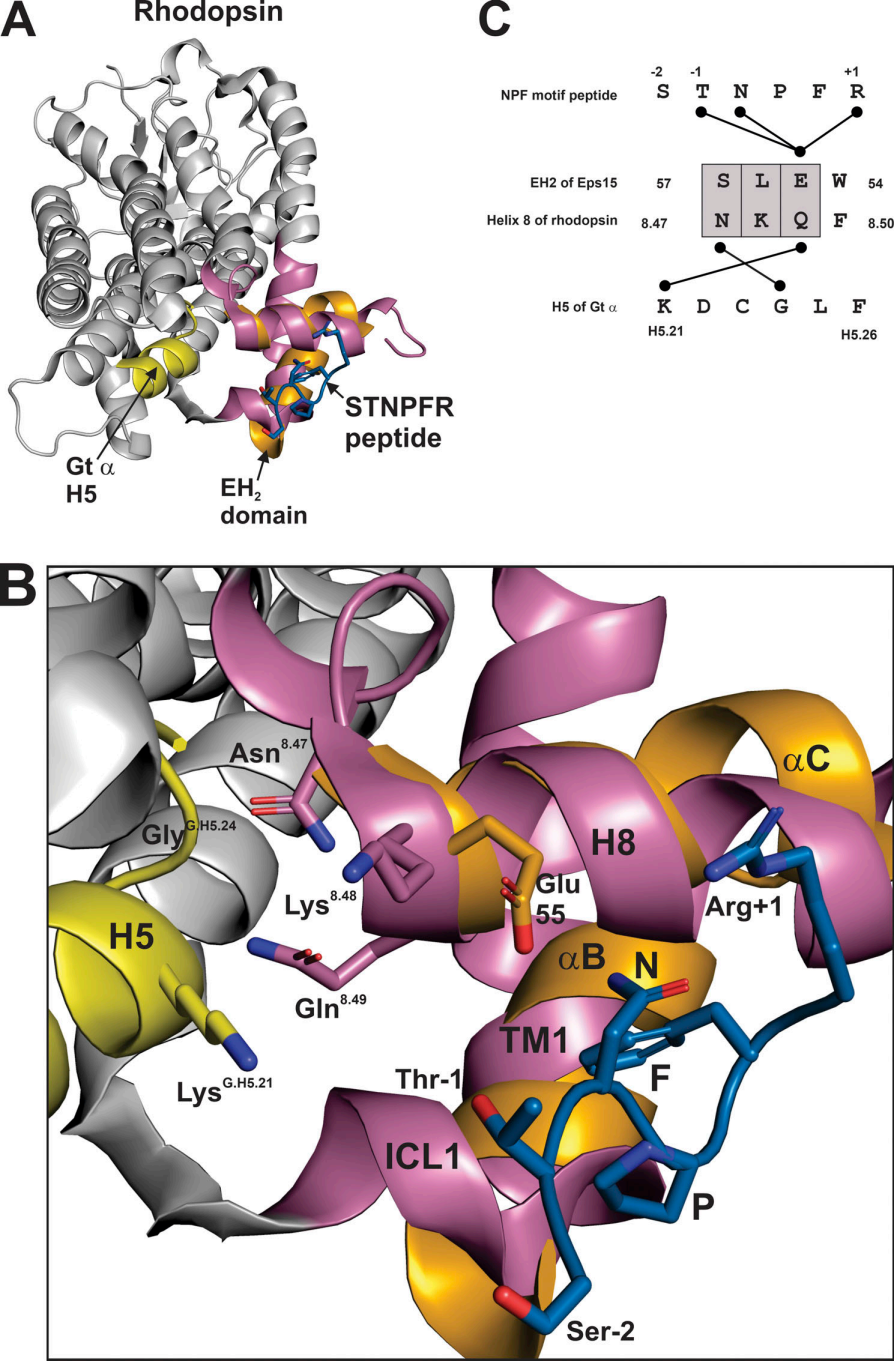
**Putative farnesyl binding site at the cytoplasmic core of rhodopsin.** **(A)** Structure of light-activated rhodopsin bound to a C-terminal Gt α peptide (yellow; PDB accession no. 3DQB), with the αB and αC helices of EH_2_ (orange) from Eps15 bound to the STNPFR peptide (blue) manually aligned with TM1 and H8 (reddish-purple) of rhodopsin. **(B)** Closeup of A showing critical side chains; the NPF containing peptide is a model of how the NPF motif of Gγ may interact with GPCRs. **(C)** Alignment of three residues, SLE, in the αC helix of EH_2_ domain with NKQ of H8 in rhodopsin (grey boxes). Interactions of SLE with the STNPFR peptide and NKQ with H5 of Gt α are indicated with black lines. PyMOL was used to determine interactions as contacts <4 Å.

A prenyl binding site on rhodopsin can be inferred from the re-examination of biochemical evidence in light of the spatial proximity of the putative NPF binding site to the Gt α C-terminal peptide-binding site. Both C-terminal Gt α and prenylated Gγ_1_ peptides can stabilize the light-activated MII state of rhodopsin (Kisselev et al., 1994; Kisselev et al., 1999). The crystal structure of light-activated rhodopsin bound to the C-terminal Gt α peptide (Scheerer et al., 2008) sheds light on how Gt α can stabilize the MII state of rhodopsin; however, no structure exists that details the interactions between rhodopsin and the prenylated Gγ_1_ C-terminus. Fig. 6 illustrates the structure of light-activated rhodopsin bound to the Gt α C-terminal peptide (PDB accession no. 3DQB), with the peptide ensconced in a pocket formed by the reorganization of TM helices from the ground state structure (PDB accession no. 1U19), most notably the outward movement of TM6. To visualize how the C-terminal Gγ_1_ peptide may interact with rhodopsin, the interaction of the αB and αC helices of the EH_2_ domain of Esp15 and the STNFPR peptide (PDB accession no. 1F8H) was superimposed on H8 and TM1 of the structure of rhodopsin activated by the Gt α C-terminal peptide (Fig. 6 A), with the area enlarged showing the side chain detail in Fig. 6 B. The relationship between the binding sites of the Gt α peptide and the STNFPR peptide is consistent with the experimental result that demonstrated the replacement of just three residues from rhodopsin (Asn^8.47^, Lys^8.48^ and Gln^8.49^) with the analogous residues from the β_2_AR abrogated binding of both Gα and Gγ C-terminal peptides (Ernst et al., 2000). Both Asn^8.47^ and Gln^8.49^ of rhodopsin make contact with the Gt α peptide (Fig. 6 C), while Glu55 of the EH_2_ domain of Esp15, analogous to Gln^8.49^ of rhodopsin, makes contact with Thr^−1^, Asn, and Arg^+1^ of the NPF-containing peptide (Fig. 6 C). The paradoxical result that replacing nine additional residues in H8 of rhodopsin (8.50–8.58) with analogous residues from the β_2_AR restored Gγ_1_ C-terminal binding to rhodopsin (Ernst et al., 2000) can be reconciled with the idea that H8 of GPCRs, with TM1 and ICL1, form an NPF binding module that may be specific for each GPCR. The spatially distinct nature of the binding sites for the NPF containing STNPFR peptide and the Gt α C-terminal peptide (Fig. 6 B) is further supported by the fact that a Gγ_1_ C-terminal peptide containing the NPF motif, but lacking the farnesyl moiety, could not inhibit the interaction between light-activated rhodopsin and Gt (Kisselev et al., 1994). The fact that the same Gγ_1_ peptide containing the farnesyl moiety could inhibit the interaction between light-activated rhodopsin and Gt (Kisselev et al., 1994) suggests that the farnesyl moiety and the Gt α C-terminal peptide have overlapping binding sites in the intracellular core of rhodopsin.

The concept of the intracellular core of the GPCR as a lipid-binding site was supported by the structure of the antagonist bound A_2A_AR (PDB accession no. 5IU8) solved by LCP crystallization, in which the lipid compound heptane-1,2,3-triol was observed in the intracellular core (Segala et al., 2016). Further, the cryo-EM structure of the GPR97-G_O_ complex was notable in that cys351^G.H5.23^ of G_O_ α was palmitoylated, a lipid modification that resided along with H5 of G_O_ α in the GPR97 intracellular core (Ping et al., 2021). Since G protein binding to this region of GPCRs is known to be allosterically linked to the ligand-binding pocket (De Lean et al., 1980), it is perhaps not surprising that a geranylgeranylated C-terminal Gγ_5_ peptide was demonstrated to decrease the affinity of the M2 receptor for the agonist carbachol and stabilize a unique conformational state of the receptor (Azpiazu and Gautam, 2006). The geranylgeranyl moiety may elicit this effect via inhibition of the movement of the GPCR helices required to attain a high-affinity agonist bound state.

### Gγ_1_–rhodopsin interactions suggest an alternative Gβγ–GPCR orientation

**Figure 7.**
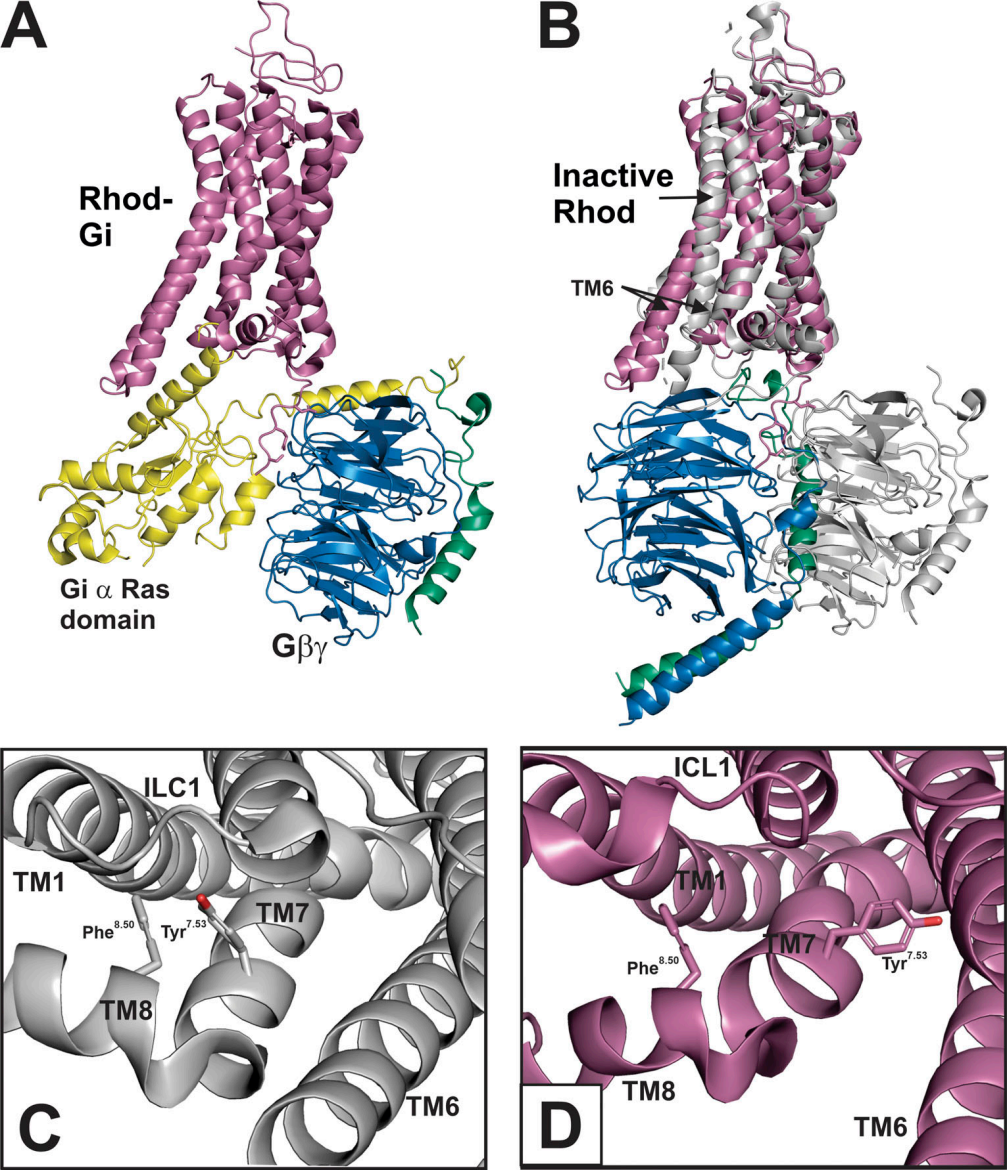
**Docking model of initial interaction of Gβγ with rhodopsin.** **(A)** Cryo-EM structure of rhodopsin (reddish-purple) bound to a heterotrimeric G protein from Tsai et al. (2019; PDB accession no. 6QNO) composed of G_i1_ α (Ras domain in yellow), Gβ_1_ (blue), and Gγ_1_ (bluish-green). **(B)** Initial docking model showing possible orientations of Gβγ from the GRK-Gβ_1_γ_2_ structure (PDB accession no. 1OMW), with respect to ground state of rhodopsin (grey; PDB accession no. 1U19), which was aligned with the G-protein-bound state of rhodopsin in A using PyMOL. The Gβγ from the GRK-Gβ_1_γ_2_ structure was used since much of the Gγ_2_ C-terminus is resolved (Lodowski et al., 2003). Gβγ from A is shown in grey for reference and G_i1_ α from A is removed for clarity. There is not enough information to know the exact location of a Gβγ docking conformation, resulting in an approximate manual docking of Gβγ in a position similar to G_i1_ α in A that would allow interactions between the C-terminus of Gγ and rhodopsin. **(C)** View of bond between Tyr^7.53^ and Phe^8.50^ in ground state rhodopsin (PDB accession no. 1U19) that is a hallmark of an inactive GPCR. **(D)** View of separation of Tyr^7.53^ and Phe^8.50^ in rhodopsin bound to G_i1_ (PDB accession no. 6QNO).

The interactions that this model predicts between the NPF motif and the prenyl moiety of Gγ and GPCR are not compatible with the structure of the rhodopsin–G_i1_ complex (Fig. 7 A), as well as other GPCR–G protein complexes. For example, the side chain of proline in the NPF motif of Gγ_1_ in the rhodopsin–G_i1_ structure (Tsai et al., 2019) is ∼40 Å away from Cys316^8.53^ of rhodopsin, which was predicted to be part of the binding site for the farnesylated C-terminus of Gγ_1_ (Downs et al., 2006). To reconcile interactions of both Gα and Gβγ with the receptor, Kisselev et al. (1999) proposed a two-step sequential fit mechanism, in which Gβγ initially and transiently occupies the space generally taken by the Gα subunit in GPCR–G protein complexes, resulting in increased efficiency of Gα coupling to GPCR. This initial conformation of Gβγ with GPCR is modeled in Fig. 7 B, which shows that the activated rhodopsin and Gβγ, in Fig. 7 A, superimposed with the structure of ground-state rhodopsin (PDB accession no. 1U19) and a possible docking orientation of Gβγ with respect to the ground state rhodopsin. In the initial docking model, the Gβγ dimer is ∼35 Å closer to ICL3 of rhodopsin, which is consistent with a crosslinking study that observed interactions between the C-terminus of Gβ with ICL3 of the α_2A_ adrenergic receptor (Taylor et al., 1996). Further, the phenylalanine in the NPF motif of Gγ_1_ was found to crosslink to a region of rhodopsin comprised of the cytoplasmic end of TM4 and IL2 (Chen et al., 2010). These crosslinking results and the proposed mechanism for the interaction between rhodopsin and the farnesylated C-terminus of Gγ_1_ are consistent with the two-step sequential fit mechanism.

### Activating features of GPCRs affect Gβγ binding

The model of the alternative of GPCR–Gβγ orientation discussed above is limited in that rhodopsin is likely not in the fully active conformation, as it has yet to bind the Gt α C-terminus. However, it is also not in the ground state, as some features of the photoactivated rhodopsin must be present for Gβγ–rhodopsin interaction, as farnesylated Gγ_1_ peptides stabilize the active form of rhodopsin (Kisselev et al., 1994), and photoactivated rhodopsin was required for induction of a conformational switch in a C-terminal Gγ_1_ peptide (Kisselev and Downs, 2003). Thus, some partially activated intermediate of rhodopsin is likely required for interactions with Gβγ; this has been observed experimentally as Gt α and Gtβγ bind to distinct conformations of photoactivated rhodopsin (Downs et al., 2006). One activating structural feature of GPCRs that would seem to be important for the rhodopsin–Gγ_1_ interaction is the conformation of the NPxxY motif in TM7 (Fritze et al., 2003), where Tyr306^7.53^ interacts with Phe313^8.50^ of H8 in the inactive state (Fig. 7 C). In the active state of rhodopsin, the bond between Tyr306^7.53^ and Phe313^8.50^ is broken (Fig. 7 D); this has significance, as Trp54 in the αC helix of the EH_2_ domain, analogous Phe313^8.50^, is critical for binding to the NPF (Fig. 4 E).

**Figure 8.**
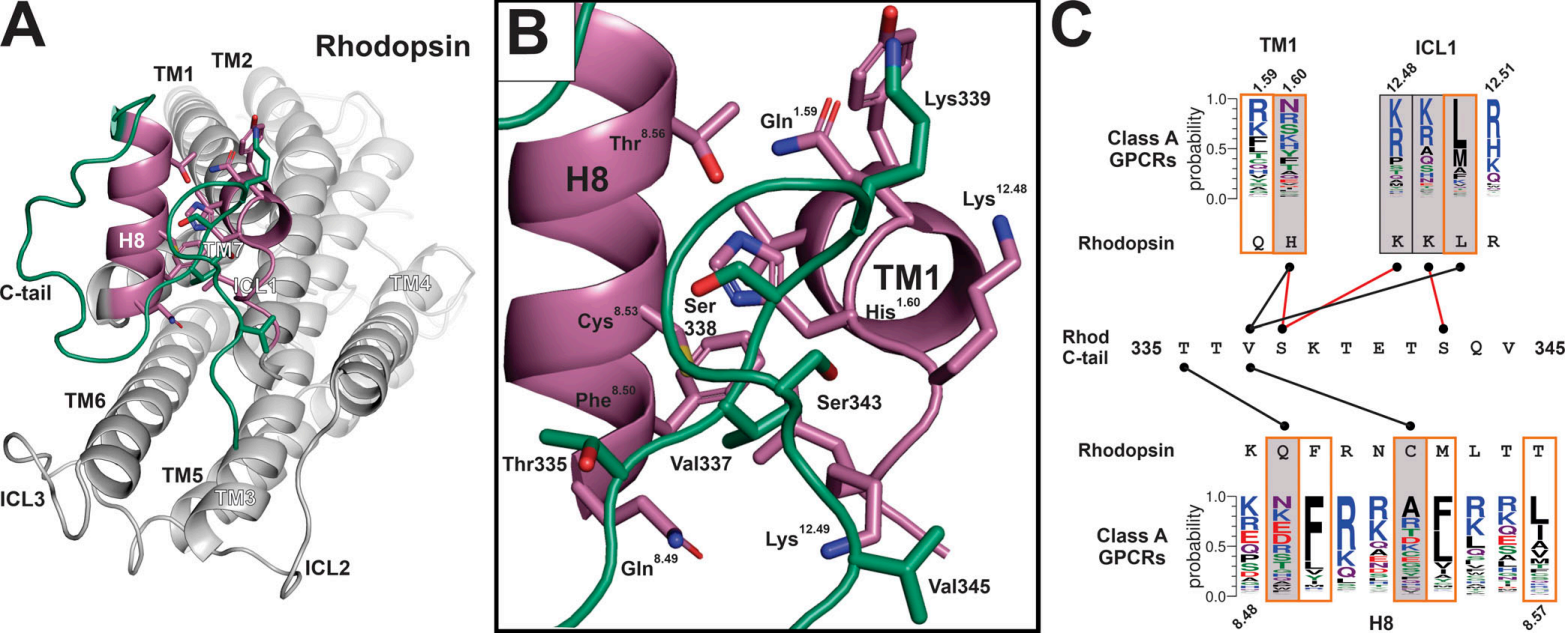
**The rhodopsin C-tail as a model for the regulation of GPCR–Gβγ interactions.** **(A)** Structure of the ground state of rhodopsin (PDB accession no. 1U19) emphasizing the interaction between TM1, ICL1, and H8 (reddish-purple), and the C-tail (green). **(B)** Closeup of A with detail of critical side chains. **(C)** Interactions of the C-tail of rhodopsin 335–345 with TM1, ICL1, and H8 of rhodopsin; above TM1 and ICL1 and below H8 are graphical representations of the sequence alignment of regions analogous to rhodopsin in Class A GPCRs. Grey boxes indicate residues in rhodopsin that interact with the C-tail; boxes with orange outlines represent residues in rhodopsin that are analogous to residues in the EH_2_ domain of Eps15 that interact with the NPF motif. Red connecting lines highlight interactions with residues in the C-tail of rhodopsin that are known to be phosphorylated.

Another activating feature of rhodopsin that may be necessary for interactions with the C-terminus of Gγ_1_ involves the conformational dynamics of the C-tail. Fig. 8 A shows the inactive form of rhodopsin (Okada et al., 2004) and how the C-tail of rhodopsin occludes the putative NPF binding site. These interactions are shown in more detail in Fig. 8 B, which reveals that the NPF binding site overlaps with the C-tail binding site. Fig. 8 C shows the interactions between the rhodopsin C-tail and TM1, ICL1, and H8 of rhodopsin, which are indicated by grey boxes. Residues in TM1, ICL1, and H8 that are analogous to the residues in the EH_2_ domain (Fig. 4) and that interact with the NPF motif are surrounded by orange boxes, demonstrating the overlap between C-tail and NPF binding sites. Since agonist bound GPCR was observed to induce conformational changes in H8 and ICL1 of the μopioid receptor in what was proposed to be an initial event in GPCR–G protein coupling (Sounier et al., 2015), it is possible that activation-dependent conformational changes in H8 and ICL1 make the binding of the C-tail to the NPF binding site less favorable.

This inhibitory role of the GPCR C-tail was proposed in a study by Pankevych et al. (2003), which found that truncation of the last 18 residues in the C-tail of the A_1_ adenosine receptor enhanced signaling. Importantly, there are no phosphorylation sites in the 18-residue region of the A_1_AR C-tail, eliminating the confounding effects of reduced activity due to arrestin binding; thus, the authors hypothesized that the C-tail reduced GPCR activity by binding to a G protein docking site on H8. The large diversity in both length and sequence of GPCR C-tails suggests that the modulation of GPCR–Gβγ interactions by the C-tail may vary among GPCR families.

In the structure of the rhodopsin G_i1_ complex (Tsai et al., 2019), the C-tail of rhodopsin bound to the surface of Gβ, as well as G_i1_ α, suggesting that Gβγ may facilitate the removal of the C-tail of rhodopsin from the NPF binding site, while also serving as a GPCR bound scaffold that can position Gβγ to maximize the efficiency of the GPCR–G protein coupling. The location of phosphorylation sites also supports the hypothesis that the C-tail may inhibit access to the NPF binding site in rhodopsin. Both Ser338 and Ser343 have been shown to be phosphorylation sites in rhodopsin (Ohguro et al., 1996), and phosphorylation of these residues has been shown to reduce the signaling activity in reconstitution experiments with purified proteins (Arshavsky et al., 1985). Since Ser338 and Ser343 of the rhodopsin C-tail are involved in contacts with ICL1 (Fig. 8 C), one explanation for this result, in the absence of arrestin binding, is the increased affinity of phosphorylated Ser338 and Ser343 for the basic residues in ICL1 at positions 12.48 and 12.49, respectively, which are conserved in class A GPCRs (Fig. 8 C).

Taken together, these activating features that allow initial interactions between GPCR and Gβγ may be related to a pre-coupled state that was suggested to be important for recognition between A_2A_AR and Gs (Huang et al., 2021). However, the initial interaction between Gγ and rhodopsin in this model does not account for GPCR–Gα contacts, which are also important for recognition; this suggests that other pre-coupled conformations exist.

### A mechanism for positive cooperativity in Gt activation by rhodopsin dimers

**Figure 9.**
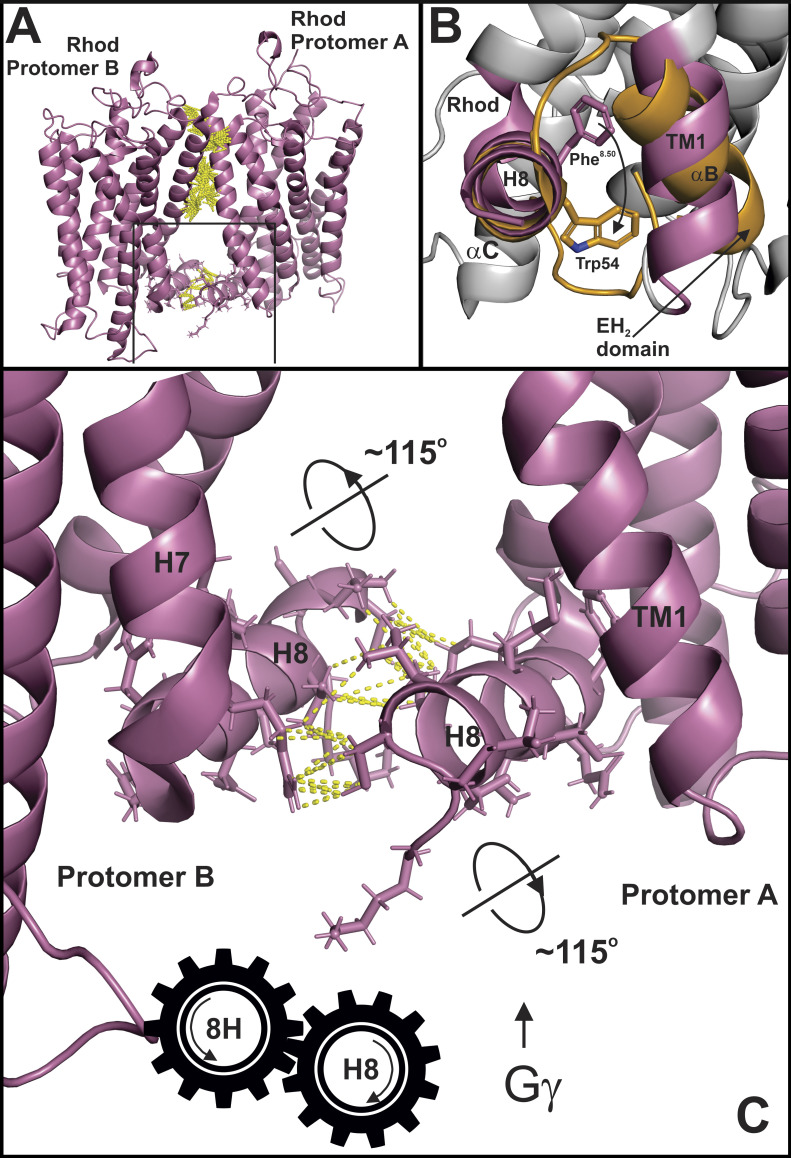
**A model for positive cooperativity Gt activation of rhodopsin oligomers.** **(A)** Structure of rhodopsin dimer (PDG accession no. 6OFJ) showing contacts between protomers as dashed yellow lines. **(B)** View of EH_2_ domain and rhodopsin as in Fig. 5 A showing the rotation required for H8 to approximate the position of the αC helix of EH_2_. **(C)** Model showing how rotation of H8 in one rhodopsin protomer would affect rotation of H8 in other protomer.

It has been observed that oligomeric forms of rhodopsin were more efficient than monomeric rhodopsin in the activation of Gt (Fotiadis et al., 2006). This could suggest an alternative mode of rhodopsin–Gt coupling (Filipek et al., 2004), in which both Gt α and Gtβγ simultaneously interact with rhodopsin protomers in an oligomeric structure; however, this positive cooperativity is also compatible with the model proposed here. An important feature of the cryo-EM structure of a rhodopsin dimer in nanodiscs was the interface, mediated by TM1 and H8 (Zhao et al., 2019); of particular importance is the extensive interactions maintaining the H8 of one protomer in an antiparallel orientation with respect to the H8 of the other protomer (Fig. 9 A). As discussed in Fig. 4, H8 of rhodopsin would need to rotate ∼115° clockwise as viewed from the distal end of H8 to most closely approximate the αC conformation of EH_2_ (Fig. 9 B). In the context of a rhodopsin dimer, the rotation of H8 in protomer A to accommodate binding of the NPF motif of Gγ could act as a gear, where the interacting residues in each H8 are the teeth, to produce a similar rotation in H8 of protomer B due to the antiparallel H8 interface (Fig. 9 C). This allosterically induced rotation in H8 of protomer B would likely initiate structural changes consistent with GPCR activation, such as the loss of the Tyr306^7.53^–Phe313^8.50^ bond, along with increasing the accessibility of the NPF binding pocket for binding Gβγ, hastening the activation process. This model could also explain the cooperative activation, or transactivation, observed in the luteinizing hormone receptor (LHR), where a signaling deficient LHR mutant was shown to activate a ligand-binding deficient LHR mutant through dimerization (Rivero-Muller et al., 2010).

### Allosteric regulation of the prenyl moiety mediated by GPCR–Gβ interactions

In the rhodopsin–G_i1_ structure (Fig. 7 A), Gβ was observed to interact with the C-tail of rhodopsin, which was posited to serve as a signaling scaffold, as it could also interact with Gα and arrestin (Tsai et al., 2019). The residues in Gβ_1_ that the C-tail of rhodopsin interacts with, Cys271, Asp290, Asp291, and Arg314, are part of a conformationally dynamic region; three of these residues, Asp290, Asp291, and Arg314, interact with phosducin (Loew et al., 1998), which facilitates the conversion of Gβγ into the tense, or T-state, as opposed to the resting, or R-state, which is the more typically observed conformation, found in structures of Gβγ bound to Gα subunits as well as in GPCR–G protein structures containing Gβγ. One distinguishing characteristic of the T-state of Gβγ is a prenyl binding pocket that forms between blade 6 and 7 of Gβ_1_ (Loew et al., 1998); binding of the Gγ prenyl moiety in this pocket decreases the hydrophobicity of Gβγ (Lukov et al., 2004), which facilitates translocation of Gβγ into the cytosol, an event that occurs upon GPCR activation of G protein (O’Neill et al., 2012).

**Figure 10.**
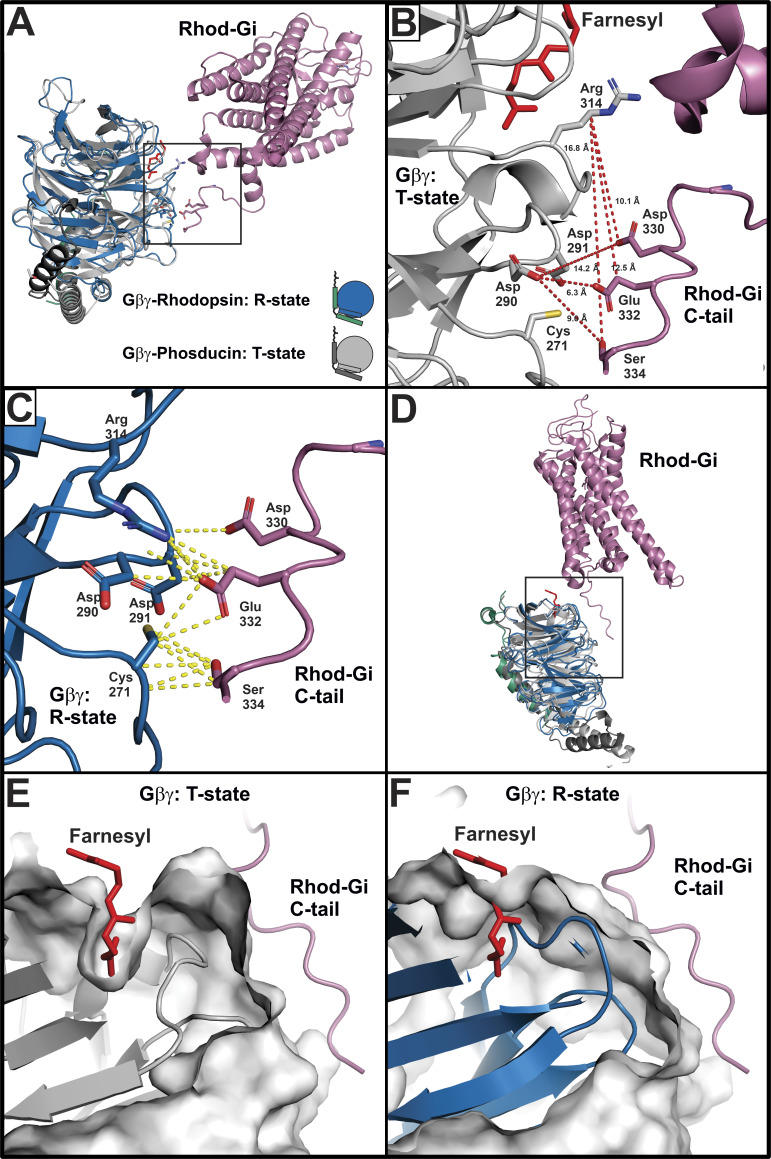
**GPCR C-tail allosterically modulates Gβγ to facilitate prenyl-GPCR interactions.** **(A)** Rhodopsin (reddish-purple) and the R-state Gβ_1_γ_1_ (blue) from the rhodopsin–G_i1_ structure in Fig. 6 (PDB accession no. 6QNO) with the T-state Gβ_1_γ_1_ (grey) from the phosducin-Gβγ structure (PDB accession no. 1A0R) superimposed on Gβ_1_γ_1_ from the rhodopsin–G_i1_ structure. **(B)** Closeup of boxed area in A showing the phosducin bound form of Gβ_1_γ_1_ (T-state). Distances between residues in the T-state conformation of Gβ_1_γ_1_ that appear to be too far for the interactions observed in the rhodopsin–G_i1_ structure are indicated by dashed red lines. **(C)** Closeup of boxed area in A showing the rhodopsin bound form of Gβ_1_γ_1_ (PDB accession no. 6QNO), with atomic distances between the C-tail of rhodopsin and Gβ_1_ <4 Å indicated by dashed yellow lines. **(D)** Rhodopsin and Gβ_1_γ_1_ from the rhodopsin–G_i1_ structure (PDB accession no. 6QNO), emphasizing the location of the farnesyl moiety (red) and the prenyl binding pocket in Gβ_1_ in the phosducin Gβγ structure, in relation to the C-tail of rhodopsin. **(E)** Close-up of boxed area in D showing the cartoon structure and the surface representation of phosducin Gβ_1_γ_1_ in the T-state, with the surface clipped to emphasize the farnesyl moiety in the prenyl binding pocket. **(F)** Close-up of boxed area in D showing the cartoon structure and the surface representation of the rhodopsin bound Gβ_1_γ_1_ from Fig. 6 in the R-state. The farnesyl moiety from the phosducin Gβ_1_γ_1_ structure is also included to show its relative position and emphasize that the surface representation of R-state of Gβ_1_γ_1_ is incompatible with prenyl binding, as the pocket is effectively eliminated with the conformational change from the T- to R-state.

Fig. 10 A illustrates the interaction of the C-tail of rhodopsin with Gβ from the rhodopsin–G_i1_ structure (Fig. 7 A), with G_i1_ α removed and Gβγ from the phosducin-Gβγ structure (PDB accession no. 1A0R) aligned with Gβγ from the rhodopsin–G_i1_ structure. A detailed view of the Gβ_1_ side chains Cys271, Asp290, Asp291, and Arg314 from the phosducin–Gβγ structure illustrates that the T-state of Gβγ is not compatible with the rhodopsin–Gβ interactions observed in the rhodopsin–G_i1_ structure (Fig. 10 B). In contrast, Fig. 10 C shows the same Cys271, Asp290, Asp291, and Arg314 residues from Gβ_1_ in the R-state, making interactions with the C-tail of rhodopsin. Fig. 10 D illustrates a different orientation of Fig. 10 A, with emphasis on the relationship between the C-tail of rhodopsin and the prenyl binding site of Gβ_1_. A closeup of Fig. 10 D with the structure and surface map of phosducin-Gβγ in the T-state reveals the prenyl binding pocket (Fig. 10 E). The elimination of the prenyl binding pocket is shown in Fig. 10 F, which includes the structure and surface map of Gβγ, from the rhodopsin–G_i1_ structure, in the R-state. The implication of these conformational changes between the R- and T-states of Gβγ is that the C-tail of rhodopsin can bind to and stabilize the R-state of Gβγ. This may be physiologically important, as cytosolic Gβγ dimers in the T-state likely need to transition to the R-state to make the prenyl moiety available for interactions with GPCR as well as the membranes. As a side note, this mechanism may also be relevant to Gβγ dependent translocation of effectors such as GRK2 to the plasma membrane (Ribas et al., 2007), as GRK2 was observed to interact with the conformationally dynamic Asp290, Arg314, and Trp332 of Gβ_1_ in the R-state (Lodowski et al., 2003), suggesting a GRK2-β_1_γ_2_ complex would have the Gγ prenyl moiety available for interactions with the membrane. Although these conformational changes in Gβγ would account for some of its signaling properties, ascribing proteins such as rhodopsin and GRK2 the ability to alter the conformation of Gβγ, as phosducin does, is highly speculative.

Evidence that the C-tails of other GPCRs interact with the same region of Gβ was seen in the structure of the M1 muscarinic receptor in complex with G_11_ (Maeda et al., 2019). Although the density in the map did not allow details of specific side-chain interactions, the C-tail of the M1 receptor was in approximately the same conformation as the C-tail of rhodopsin in the rhodopsin–G_i1_ structure (Fig. 7 A). The authors of the M1R-G_11_ structure suggested that the C-tail interactions with Gβ occurred at an earlier intermediate step in GPCR–G protein coupling; this is compatible with the initial docking model of GPCR and Gβγ, and suggests the C-tail may allosterically modulate Gβγ to expose the prenyl moiety for more favorable interactions with GPCR.

### A mechanism on how Gβγ couples Gα to GPCR

The C-terminus of Gγ_1_ was proposed to be masked in the Gβγ complex, and interaction with light-activated rhodopsin was suggested to induce a conformational switch in the Gγ_1_ C-terminus, allowing it to make high-affinity interactions with the receptor (Kisselev et al., 1995b). Details of the conformational switch at the C-terminus of Gγ_1_ were revealed by the solution structure of a C-terminal farnesylated decapeptide from Gγ_1_ in the presence of rhodopsin (Kisselev and Downs, 2003). Light activated rhodopsin, but not inactive rhodopsin, was able to promote the dynamic transition of the farnesylated Gγ_1_ decapeptide from random coil to α helix, facilitated by an intermediate 3^10^ helix. Kisselev and Downs predicted that the conserved NPF motif in Gγ isoforms acted as a proline switch that could be stabilized by activated rhodopsin, allowing formation of the Gγ C-terminal α helix (Kisselev and Downs, 2003). The putative NPF binding site in rhodopsin is consistent with this prediction; it also adds a mechanistic explanation, as the Asn-Pro β-turn conformation the NPF motif acquires in binding rhodopsin has been shown to be a critical step in helix nucleation, with short 3^10^ helices serving as intermediates (Pal et al., 2003).

**Figure 11.**
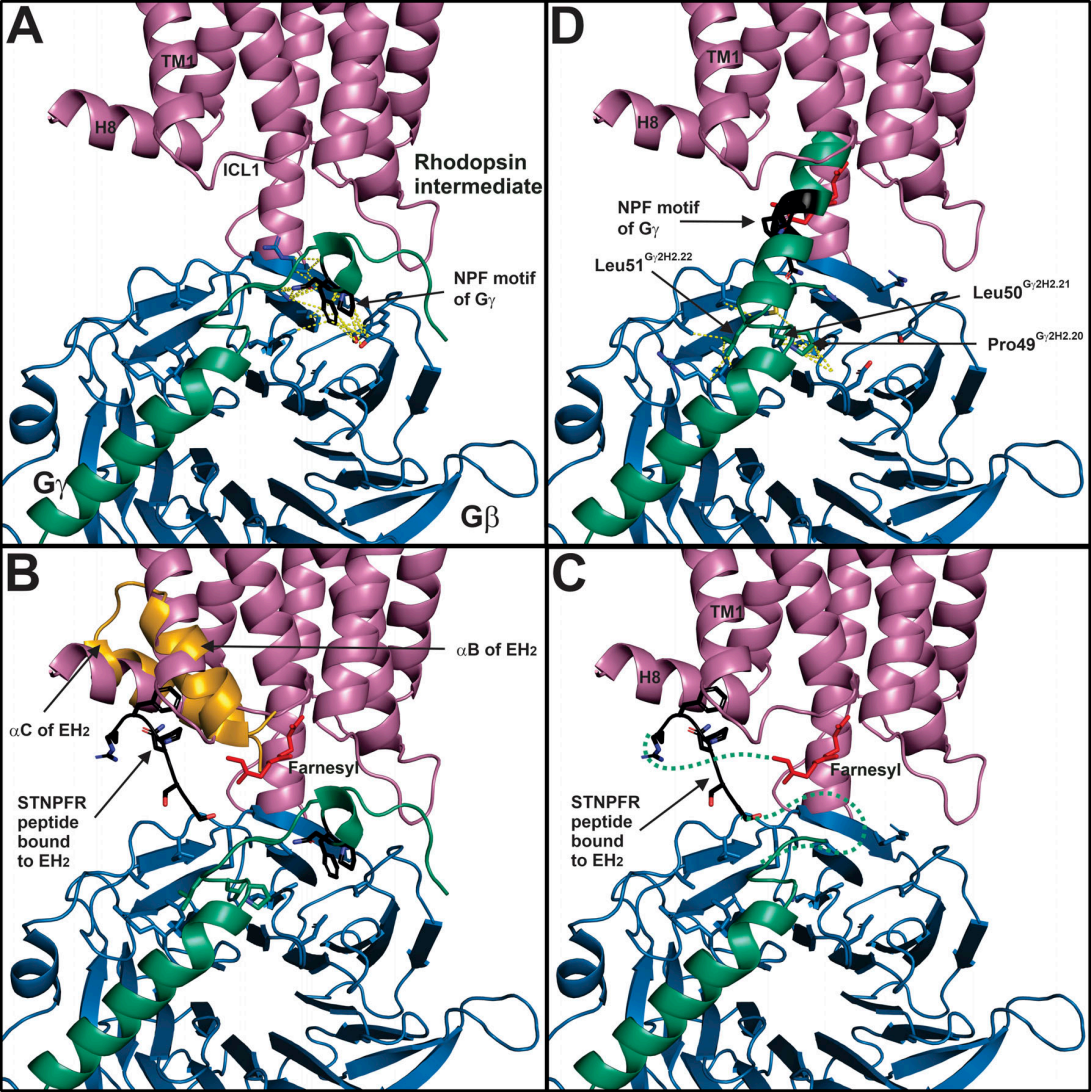
**Activation mechanism for the Gγ C-terminal conformational switch.** **(A)** An initial docking model of the ground state of rhodopsin (PDB accession no. 1U19) and Gβ_1_γ_2_ from the Gβ_1_γ_2_–GRK2 complex (PDB accession no. 1OMW) was constructed as described in Fig. 7 B, emphasizing atomic distances within 4 Å between the NPF motif (black) in Gγ_2_ (bluishgreen) and Gβ_1_ (blue), with dashed yellow lines. The C-tail of rhodopsin was removed for clarity. **(B)** Same view as A, except the STNPFR peptide (black) bound to αB and αC helices of EH_2_ (orange) from Eps15 was manually aligned onto TM1 and H8 of rhodopsin. **(C)** Same view as B except the αB and αC helices of EH_2_ were removed to illustrate the STNPFR peptide as a surrogate for Gγ NPF motif binding to rhodopsin. In this conformation, the Gγ_2_ C-terminus is unmasked and the NPF motif no longer interacts with Gβ. A farnesyl moiety (red) is modeled in the cytoplasmic core of rhodopsin to represent one of the initial interactions between rhodopsin and Gγ_1_. **(D)** Same view as C, except an α helix was constructed in PyMOL based on the C-terminal h2 region of Gγ_1_: KGIPEDKNPFKELKGGC, and modeled as a helical extension of the three residues of the H2 Gγ_2_ helix, Pro49^H2.20^, Leu50^H2.21^, and Leu51^H2.22^, that make extensive contacts with Gβ (dashed yellow lines). The helical h2 region of Gγ_1_ is in bluishgreen, except for the NPF motif, which is black. There is not enough information to know exactly where the prenyl binding site is in the hydrophobic core of rhodopsin, thus the location of the farnesyl moiety, which would direct the extension of the Gγ h2 helix into the core of the receptor, is somewhat arbitrary.

Fig. 11 details how the rhodopsin-induced conformational switch in Gγ_1_ could catalyze Gα coupling to GPCR. In Fig. 11 A, the C-terminus of Gγ_1_ is unavailable for interactions with the ground state of rhodopsin, as the NPF motif (black) is embedded in the Gβ subunit. Fig. 11 B compares the NPF motif of Gγ_1_ in the masked state with the STNPFR peptide bound to the EH_2_ domain of Eps15, superimposed onto H8 and TM1 of rhodopsin. It is conceivable that the Gγ_1_ C-terminus samples the random coil state, anchored to rhodopsin by the NPF motif, like the STNPFR peptide. Thus, there may be an additional interaction that facilitates the unmasking to allow the subsequent binding of the NPF motif to rhodopsin; alternatively, part of the binding mechanism of the rhodopsin NPF binding site may be to extract the NPF motif from Gβ. The proposed location of the farnesyl moiety in the intracellular core of rhodopsin is shown relative to the location of the proposed NPF binding site in rhodopsin (Fig. 11 C). The model presented here predicts that the rhodopsin NPF binding site facilitates the transition of the Gγ_1_ C-terminus into an α helix, possibly via the formation of a type I Asn-Pro β-turn. Without the NPF motif of Gγ binding to Gβ, two principal interactions anchor the N- and C-terminal ends of the h2 domain of Gγ in the initial docking model of the GPCR–Gβγ complex. Preceding the N-terminus of the h2 domain, Gγ residues at the C-terminus of the H2 domain (Fig. 2) at positions H2.20, H2.21, and H2.22 make extensive interactions with Gβ, forming the beginning of an α helix (Fig. 11 D); at the C-terminal end of the h2 domain, the farnesyl moiety of Gγ_1_ bound to the intracellular core of rhodopsin provides the second anchor point for the h2 domain. The result of the transition of the h2 domain of Gγ_1_ from a disordered state to an α helix would produce an outward force along the axis of the α helix, toward the residues at positions H2.20, H2.21, and H2.22 in the Gγ H2 domain, and in the opposite direction toward the farnesyl moiety in the intracellular core of rhodopsin. With Gβγ stabilized by the C-tail of rhodopsin (Fig. 7 A), a force from the α helix transition would be directed toward the intracellular core of rhodopsin, providing a mechanism for the outward movement of TM6, and the creation of a favorable binding site for the C-terminus of Gt α. This mechanism is also consistent with the observation that the whole Gβγ protein, not just the prenylated C-terminal Gγ peptide, was required to couple Gα to GPCR (Kisselev et al., 1999). Since H8 and TM1 have been shown to be dynamic during the initial stages of GPCR–G protein coupling (Sounier et al., 2015), the activation process may destabilize the NPF binding pocket in rhodopsin and hasten the release of the Gγ_1_ C-terminus and subsequent binding of the Gt α C-terminus.
